# Prevalence and determinants of dietary practices among pregnant women in eastern Ethiopia

**DOI:** 10.1186/s40795-021-00494-4

**Published:** 2022-01-11

**Authors:** Meseret Belete Fite, Abera Kenay Tura, Tesfaye Assebe Yadeta, Lemessa Oljira, Kedir Teji Roba

**Affiliations:** 1 Department of Public Health, Institute of Health Sciences, Wollega University, Nekemte, Ethiopia; 2 School of Nursing and Midwifery, College of Health and Medical Sciences, Haramaya University, Harar, Ethiopia; 3 Department of Obstetrics and Gynaecology, University Medical Centre Groningen, University of Groningen, Groningen, the Netherlands; 4 School of Public Health, College of Health and Medical Sciences, Haramaya University, Harar, Ethiopia

**Keywords:** Dietary practice, Dietary diversity, Food variety score, Pregnancy, Ethiopia

## Abstract

**Introduction:**

Appropriate dietary practices in pregnancy are critical to meet the increased metabolic and physiological demands; however, information about dietary practices among pregnant women, particularly rural residents, is limited. The study aimed to assess the level of appropriate dietary practices and associated determinants among pregnant women in Haramaya District, eastern Ethiopia, 2021.

**Methods:**

A community-based cross-sectional study was conducted among 448 pregnant women in Haramaya District, Eastern Ethiopia. Data was collected through face-to-face interviews by trained research assistants, using a validated frequency questionnaire. The pregnant women were labeled as “appropriate dietary practice” when they consumed at least four meals daily, had a good food variety score, high dietary diversity score, and high consumption of animal source foods during the reference period. Otherwise, they were defined as “inappropriate.” A Poisson regression model with robust variance estimation was used to investigate the association of the independent variables with the dietary practice. An adjusted prevalence ratio with a 95% confidence interval was reported to show an association using a *p*-value < 0.05.

**Results:**

The appropriate dietary practice among the study participants was 15.2% (95% CI = 12–18%). Of the respondents, 29.46, 37.5, and 24.7% had a high dietary diversity, high food variety score, and high consumption of animal source foods. The appropriate dietary practice was more prevalent among merchant women (APR = 2.07; 95% CI 1.07–4.02) and those whose husbands have at least a high school educational level (APR = 1.96; 95% CI 1.06–3.46). However, the prevalence of appropriate dietary practice was significantly lower among those who chewed khat (APR = 0.58; 95% CI 0.37–0.90) and among respondents who reported restriction of the intake of some foods (APR = 0.36; 95% CI 0.20–0.65).

**Conclusion:**

We found sup-optimal appropriate dietary practice among pregnant women in this predominantly rural setting. Additionally, the lower appropriate dietary practice was observed among women who reported chewing khat and experienced restriction of dietary consumption during pregnancy. Therefore, nutrition policy programs and interventions aimed at encouraging maternal nutritional guidance and counseling are recommended.

**Supplementary Information:**

The online version contains supplementary material available at 10.1186/s40795-021-00494-4.

## Background

A healthy and balanced diet is essential for human beings to have sustainable health and appropriate working of the body [1]. During pregnancy, there are increased metabolic and physiologic demands on the body, which are expected to be met by adequate nutrition [2]. However, at conception, many women do not have sufficient micronutrient intake to meet their bodies’ demands [3]. Therefore, this lack of specific dietary practices during pregnancy is critical for under-nutrition and micronutrient deficiencies resulting in poor birth outcomes [4]. The basis for these problematic dietary practices in unindustrialized areas include inadequate access to food sources and poor knowledge on the essence of good diet quality and quantity. Moreover, pregnant women in developing countries mainly consume a diet predominantly plant-based, macronutrient imbalanced, and consisting of inadequate micronutrients [5–8].

Accordingly, studies revealed that good diversity practices in pregnant women in low-middle income countries varied, ranging from 20% in Pakistan [9] to 70% in India [10]. However, most pregnant women in Sub-Saharan Africa have insufficient dietary consumption and do not meet the recommended dietary allowances (RDA) [11]. Likewise, evidence suggests the appropriate nutritional practices of pregnant women in Ethiopia ranged from 19.9% in Gojjam [5] to 40.1% in Gonder [6]. The researchers used various tools to evaluate the dietary practices of pregnant women; some were using nutrient content however meal frequency was the most common.

Few studies reported factors affecting maternal dietary practices in different parts of Ethiopia. For example, edible crop production, attitude, dietary knowledge [6], cultural prohibition, women’s educational level, income, and ownership of livestock [5, 11] were documented as predictors of the appropriate dietary practices. In addition, the age of the mother, food taboo, and meal frequency was explored as determinants of the dietary practices among pregnant women [12].

We hypothesized that pregnant women in this study setup exhibit low appropriate dietary practice, and this is affected by different independent predictors. Therefore, the objective of this study, is to assess dietary practice and the determinants among pregnant women.

## Methods

### Study settings

The study was embedded into the Haramaya Health Demographic Surveillance and Health Research Centre (HDS-HRC), which was established in 2018. The HDS-HRC located in Haramaya district. Haramaya District, located 500 km away from the capital city, Addis Ababa to the east. Haramaya district consists of 33 kebeles (the lowest administrative unit in Ethiopia). HDS-HRC covers 12 rural kebeles which is representative and randomly selected by considering geographic and environment issues. In HDS-HRC 2306 pregnant women were followed. The district has mixed farming, with the major cash crop being khat (*Catha edulis* Forsk) [13]. The study was conducted from January 5 to February 12, 2021.

### Study design and population

A community based cross-sectional study was conducted. All pregnant women living in the district constituted the source population; whereas all pregnant women who lived in the selected kebeles for at least 6 months during the study period were the study population. The sample size was determined using single and double population proportion formulas with their corresponding assumption, and the largest sample was considered. However, this study is part of a larger longitudinal study which obtained birth outcome information in pregnant women; thus, the sample size which was used in this study was calculated from the larger study that included 475 pregnant women. Birth outcome (Low birthweight) was the main outcome was used in calculating the sample size. Sample size was determined from Charan and Biswas study [14].

Where, n = sample size, Z α/2 = 1.96 at type 1 error of 5%, Z β = 0.84 at 80% power, P1 = LBW in pregnant women with no gestational HTN (22.9%), P2 = LBW in pregnant women with gestational HTN (10.37%), p1-p2 = difference in prevalence of low birth weight between pregnant women with no gestational HTN at birth and those with gestational HTN and p = pooled prevalence = (p1 + p2)/2 [15].. A study among pregnant women in the area reported LBW prevalence of 21.0%% (LBW DD). Hence, we proposed that LBW in pregnant women with gestational HTN would be 22.9%, while those with gestational HTN would remain 10.37%,. Hence, p1 = 22.9%, p2 = 10.37%, their proportions being p1 = 0.229 and p2 = 0.1037, and p = (0.229 +  0.1037)/2 = 0.16635.

Using the above descriptive, the sample size *n* = 2(1.96 +  0.84) 2 × 0.16635(1–0.21)/(0.229–0.1037) 2, *n* = 2.152/0.01, equal 216 was calculated, which implied we needed to recruit 216 participants in each arm of the study (half in the with gestational HTN group and a half in the no gestation HTN group) making 432 participants showing a significant association between gestational HTN and LBW. Nevertheless, by adding 10% non-response rate to 475 participants were included. After constructing a sampling frame from the HDS-HRC database, simple random sampling was applied to the eight randomly selected kebeles and then the eligible women were selected using computer generated lottery method.

### Data collection and measurement

Data was collected through interview administered questionnaires by trained research assistants. The questionnaire contained data on socio-economic, obstetric, maternal perception, food consumption, dietary knowledge, attitude, and practices of pregnant women. The questionnaire was initially prepared in the English language and was translated to the local language (Afan Oromo) by an individual with good command of both languages. It was also pre-tested on 10% of the sample in Kersa District before data collection. In addition, mid-upper arm circumference (MUAC) was measured to assess nutritional status.

Four measures were used to measure the dietary practices of pregnant women including dietary diversity, food variety, animal source foods consumption, and frequency of meals. In this context, the formerly validated food frequency questionnaire (FFQ) containing 27 of the most commonly lists of food items consumed by the district community was used to assess dietary diversity of the study participants [16–20]. Additionally, this validated FFQ was used to assess dietary diversity of the participants [21, 22]. Initially, the list of food items was established based on consultation of key informants living in the study area, who knew the culture, local language, and foods typically consumed. Then the food frequency questionnaire was pretested on 10% of the sampled pregnant women in the district who were not included in the main study and necessary modifications were made based on the observations. In addition, pretested food frequency questionnaires were carried out on 10% of the sampled pregnant women of the district not included in the main study. Necessary modifications were made before actual implementation to generate data. Finally, to measure the consumption of each food per day, per week or per month for the FFQ in the past 3 months to consider the difference of dietary consumption within a day of a week to take the concept into account. However, we considered the greater difference of dietary practice in the local community over the day of the week, the intake of each food item per day [6, 23] was not taken as a cut-off point to label consumers. In doing so, pregnant women were defined as “consumer” of a food item if they had consumed those items at least once over a period of a week [21, 24].

Validity and reliability were assessed for each construct by using the common factor analysis with oblique (Promax) rotation and were censored by the use of already validated FFQ from a similar study. The reliability or internal consistency of each scale was assessed using Cronbach‘s alpha values as the reliability estimates and ranged from 0.67 to 0.86. A Cronbach‘s alpha of 0.7 was generally considered acceptable (Nunnally, 1978). In this study, the Cronbach‘s alpha value was 0.76 as well as the test-retest method was used to determine the reliability of the instruments during the pretest. A test-retest correlation coefficient of 0.76 (CI: 0.61–0.82) was computed from the two sets of data and found to be adequate. A value of + 0.80 or greater was generally would indicate good internal consistency.

The food items in the FFQ were grouped into ten food groups. These are: cereal, white roots and tubers, pulse and legumes, nuts and seeds, dark green leafy vegetables, other vitamin A-rich fruits and vegetables, meat, fish and poultry, dairy and dairy product, egg, other vegetables, and other fruits [25]. The sum of each food group that the pregnant women consumed over a period of 1 week were calculated to analysis the dietary diversity score (DDS). Furthermore, dietary diversity score was converted into tertiles, and the highest tertile used to label “high” dietary diversity score whereas both lower tertiles combined were defined as” low” dietary diversity score. Food variety score (FVS) is the frequency of individual food items consumed in the reference period of the study. Therefore, it was estimated by the intake of 27 food items by each individual over 7 days [21],with maximum of FVS fourth. Finally, the mean FVS of pregnant women was calculated and those of them with FVS greater than the means were labeled as having “high” food variety score whereas those with FVS lower than the means were defined as having “low” FVS. Furthermore, consumption of foods from animal source (ASF) was estimated by counting the frequency of each food from animal sources that pregnant women ate over a reference period. Animal source foods score was also converted into terciles and the highest tercile used to label as “highs, while the two lower terciles combined were defined as “low” ASF.

### Data quality assurance

Two days of rigorous and extensive training with the final version of the questionnaires was given to each data collector and supervisor prior to pre-test. Collected data was checked by supervisors before being sent to the data entrée on daily basis. We pre-tested the questionnaires on 10% of the sampled pregnant women of the kersa district, that were not included in the main study, and modification was done based on the pre-test observations. The supervisors kept the alleyway of the field procedures and checked the completed questionnaires daily to approve the accuracy of data collected, and the research team managed the overall work of data collection.

### Data processing and analysis

Data were double entered using EPiData version 3.1 software. Data were cleaned, coded, and checked for missing and outliers, for further analysis exported to STATA version 14 (College Station, Texas 77,845 USA) statistical software. The outcome variable was dichotomized as dietary practice = 1 (appropriate) and dietary practice = 0 (inappropriate). Thus, the Poisson regression analysis model with robust variance estimate was fitted to identify predictors of the dietary practice of women. For multivariable analyses, only variables that displayed a *p* < 0.25 in the bivariate analyses were entered in the adjusted model. The backward regression was fitted with selected socio-economic and fertility-related variables. The results are presented as adjusted prevalence ratios (APRs) with 95% CI. The statistical level of significance was set at alpha = 5%. The explanatory variables were examined for multi-collinearity before taking them into multivariable models using correlation matrix for the regression coefficients, using the standard errors, and variance inflation factor value. Possible interactions between covariates were tested. Akaike’s information criterion (AIC) and Bayesian information criterion (BIC) were used to test for model fitness.

To estimate the economic level of the families, a wealth index was employed. The wealth dispersion was generated by applying principal component analysis. The index was calculated based on the ownership of latrine, selected household asset, quantity of livestock, and source of water used for drinking, that was to 41 household variables (Supplementary file 1). Nutritional knowledge of the women was gauged through 16 nutritional knowledge questions on the feature of nutrition needed in their course of pregnancy. Lastly, the highest tertile was defined as having “Good” nutritional knowledge and the two lower tertiles were labeled as “Poor” nutritional knowledge. The maternal attitude was evaluated with 12 Likert scale questions using PCA. The factor scores were totaled and classified into tertiles (three parts), and the highest tertile was defined as having a “Favorable” maternal attitude and the two lower tertiles were characterized as “Unfavorable” maternal attitude. The maternal perceived vulnerability of malnutrition was evaluated with 10 Likert scale questions using PCA. The factor scores were totaled and classified into tertiles (three parts), and the highest tertile was defined as having a perceived vulnerability “Yes” and the two lower tertiles were characterized as “No” maternal perceived vulnerability. Similarly, perceived severity of malnutrition, perceived benefit to healthy nutrition perceived barrier to healthy nutrition and perceived self-efficacy to control malnutrition during pregnancy were calculated by using their composite questions. Women’s autonomy was evaluated by seven validated questions which were adopted from the Ethiopian demographic health survey [22]. For each response to a question, the response to each question was coded as “one” when the decision was made by the pregnant women alone or jointly with their husband, otherwise “zero”.

### Ethical consideration

All methods of this study were carried out in accordance with the Declaration of Helsinki-Ethical principle for medical research involving human subjects. Ethical approval letter was obtained from Haramaya University Institutional Research Ethics and Review Committee (IRERC) with a reference number of (IHRERC/266/2020) before the commencement of data collection. Written informed consent to participate was obtained from participants and legally authorized representatives “of minors below 16 years of age and illiterates” and their privacy and confidentiality were maintained. All personal identifiers were excluded, and data was kept confidential and used for the proposed study only.

### Operational definition

#### Meal frequency

Is defined as how many times a day peoples eat or several daily eating occasions.

#### Appropriate dietary practices

When women had at least four meals daily, good FVS, high DDS, and high ASF consumption, whereas it was inappropriate when women had less than four meals daily or Low FVS or low DDS or low ASF consumption [6, 23].

#### Wealth index quintile

Was computed from the wealth score of the households by PCA and the composite was ranked by quantile. Quintile was used to label the household’s wealth status to poorest, poor, middle, rich, and richest category.

## Results

### Socio-demographic characteristics

A total of 475 pregnant women were eligible, 448 consented, making a response rate of 94.3%. The mean age of the women was 25.68 (+ 5.1), ranging from 16 to 36. The majority of the respondents could not read or write (73.88%), were housewives (96.1%), farmers (93%), and had a family size of 1–5 (76.56%). Only 20.09% of the respondents were in the richest quintiles, Table 1.

**Table 1:** Socio-demographic of pregnant women in Haramaya District, eastern Ethiopia, 2021 (*n* = 448)

Variables	Frequency(n)	Percentage (%)
Age (years)		
< 18	25	5.58
18–35	400	89.29
> 35	23	5.13
Mean (+ SD)	25.68(+ 5.16)	
Educational level of the woman		
Can’t read or write	331	73.88
Read or write	26	5.81
Formal education	91	20.31
Educational Level of husband		49 (23.33)
Can’t read or write	259	57.81
Read or write	61	13.62
Grade 1–8	102	22.77
Grade 9 and above	26	5.8
Occupation of the woman		
Housewives	433	96.65
Merchants	15	3.65
Occupation of husband		
Farmers	420	93.75
Daily labors	28	6.25
Family size		
1–5	343	76.56
> 5	105	23.44
Agricultural land possession		
No	271	60.49
Yes	177	39.51
Livestock possession		
Yes	299	66.74
No	149	33.26
Wealth Index (Quintile)		
Poorest	90	20.09
Poor	90	20.09
Middle	89	19.87
Rich	90	20.09
Richest	89	19.87
Parity		
0	103	22.99
1–4	294	65.63
> 5	51	11.38

### Prevalence of dietary practice

**Fig. 1:**
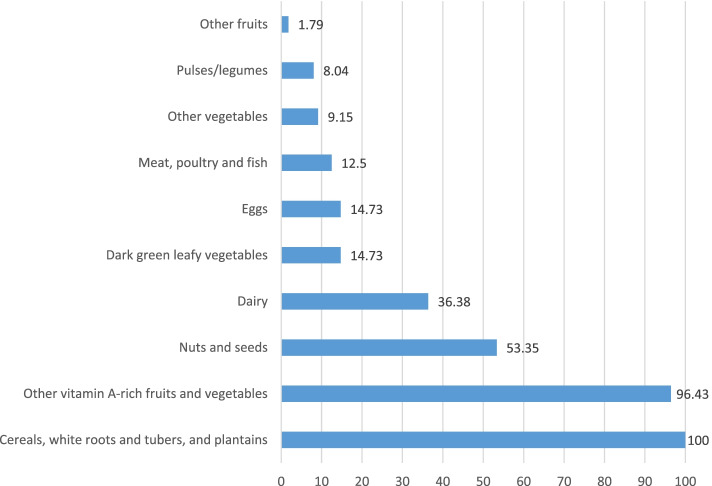
Percentage of different food groups consumed by pregnant women in Haramaya District, Eastern Ethiopia,2021

From the total respondents, 29.46% (25–34%), 37.5% mean (+ SD) (9.03 + 2.79) and 24.78% (2–29%) of them had high dietary diversity, high food variety score and high consumption of animal source food, respectively. The prevalence of the appropriate dietary practice of pregnant women in the present study was 15.2% (95% CI = 11.96–18.84), Table 2. Considering the consumption of the main food groups cereals (100%), other vitamin A-rich fruits and vegetables (96.43%), and pulses and nuts (53.35%) constitute a major part of women’s diet. Consumption of other fruits (1.7%) was minimal. Figure 1.This monotonous diet may reflect both their cultural eating habits and lower availability of a variety of foods during the survey period.

**Table 2:** Dietary practices of pregnant women in Haramaya district, eastern Ethiopia,2021

Variables	Frequency(n)	Percentage (%)
Dietary Diversity Score (DDS)		
Low	316	70.54
High	132	29.46
Mean (+ SD)	3.73(+ 1.33)	
Food Variety Sore (FVS)		
Low	280	62.50
High	168	37.50
Mean (+ SD)	9.03(+ 2.80)	
Animal Source Foods (ASFs)		
Low	337	75.22
High	111	24.78
Mean (+ SD)	25.68(+ 5.16)	
Meal frequency		
< 4	331	73.88
> 4	117	26.12
Mean (+ SD)	2.98(+ 0.84)	
Dietary practice		
Appropriate	433	96.65
Inappropriate	15	3.65

### Factors associated with dietary practice

In the bi-variable analysis, husband’s educational level, woman’s occupational status, ANC visit, perceived venerability to malnutrition, perceived severity to malnutrition, perceived benefit to healthy nutrition, khat chewing, food restriction and food aversion (strong disliked of foods) were found to be a candidate for multivariable analysis at *p* < 0.25. Level of appropriate dietary practice was higher among respondent who were merchant women (APR = 2.07; 95% CI = 1.07–4.02), those whose husbands had at least high school educational level (APR = 1.96; 95% CI = 1.06–3.46). However, level of appropriate dietary practice was significantly lower among those who reported chewing khat (APR = 0.58; 95% CI = 0.37–0.90) and restrict food taking during pregnancy (APR = 0 .36; 95% CI = 0.20–0.65), Table 3.

**Table 3:** Factors associated with dietary practice among pregnant women in Haramaya district, eastern Ethiopia, 2021

Variables	Dietary Practice		CPR (95%CI)	APR (95%CI)	*P*-value
	Appropriate (*n* = 68)	Inappropriate (*n* = 380)			
Educational Level of Husband					
Illiterate	33 (48.53)	287 (75.53)	1	1	
Elementary School	26 (38)	*76 (20.00)*	2.47 (1.554, 3.929)	1.99 (1.214, 3.280)	0.006*
High school and above	9 (13.24)	17 (4.47)	3.37 (1.806, 6.239)	1.92 (1.063, 3.460)	
Occupational status of Women					
Housewives	61 (89.71)	372 (97.89)	1	1	
Merchants	7 (10.29)	8 (2.11)	3.31 (1.837, 5.973)	2.07 (1.071, 4.016)	0.030*
ANC visit					
No	17 (25.00)	147 (38.6)	1	1	
Yes	51 (75.00)	233 (61.32)	1.73 (1.035, 2.898)	1.36 (0.776,2.394)	0.281
Perceived Venerability					
No	18 (26.47	(67 (17.63)	1	1	
yes	50 (73.53)	313 (82.37)	1.54 (0.947, 2.496)	0.82 (0.469 1.428	0.481
Perceived severity					
No	47 (69.12)	313 (82.37)	1	1	
Yes	21 (30.88)	67 (17.63)	1.83 (1.155, 2.892)	1.31 (0.747 2.303)	0.346
Perceived Benefit					
No	39 (57.35)	288 (75.79)	1	1	
Yes	29 (42.65)	92 (24.21)	2.01 (1.302, 3.099)	1.39 (0.811, 2.412)	0.228
Chat chewing					
No	38 (55.88)	142 (37.37)	1	1	
Yes	30 (44.12)	238 (62.63)	0.53 (0.341, 0.824)	0.58 (0.370, 0.901)	0.016
Drinking water					
Protected	42 (61.76)	156 (41.05)	1	1	
Unprotected	26 (38.24)	224 (58.95)	0.49 (0.312,0.771)	0.61 (0.350, 1.048)	0.073
Food restriction					
No	53 (77.94)	246 (64.74)			
Yes	15 (22.06	134 (35.26)	0.57 (0.331, 0.974)	0.36 (0.200, 0.651)	0.001**
Food aversion (Strong disliked of foods)					
No	47 (69.12)	267 (70.26)	1	1	
yes	21 (30.88)	113 (29.74)	0.96 (0.594, 1.533)	0.99 (0.631, 1.554)	0.967

Appropriate dietary practice: is defined as the consumption of at least four meals daily, high DDS, high FVS and high ASF; otherwise, Inappropriate dietary practice

Drinking water sources: ‘Protected’ if tap/hand pump and not otherwise Elementary school: Grade 1–8

*CPR* Crude Prevalence Ration, *APR* Adjusted Prevalence Ratio, *CI* Confidence Interval at 95%

CPR: was obtained from bi-variate of Poisson regression analysis model with robust variance estimate

APR, CI and *P*-Value were found from multivariable Poisson regression analysis model with robust variance estimate.

** statistically significant at *p*-value < 0.001, * statistically significant at *p*-value < 0.05

## Discussion

In the current study, the level of appropriate dietary practice among pregnant women was 15.18% and was noted to be sub-optimal. Moreover, women’s occupational status, husband’s educational level, chewing chat, and restriction of the intake of some foods were identified as predictors of dietary practice in Haramaya District. Adequate nutrition during pregnancy is essential for maternal and child health [3]. There is mounting evidence that insufficient consumption of a balanced and quality diet during pregnancy significantly affects fetus health and development and may result in poor birth outcomes [4]. At the beginning of pregnancy, many women lack sufficient micronutrient stores to meet the increased physiological requirements [1], and they are more vulnerable to malnutrition [26]. Several epidemiological studies indicated that inappropriate dietary practice contributes to maternal undernutrition and micronutrient deficiency in resource-limited countries [9, 25]. Pregnant women’s diet must supply adequate nutrients for the mother, fetus and effective lactation. Despite this reality, in this present study, noted appropriate dietary practice among pregnant women was very low. This figure was much lower than studies conducted in Illu Aba Bor Zone, Southwest Ethiopia [27], Dessie town, northeastern Ethiopia [28], and Guto Gida district, western Ethiopia [29]. The result of this study has modest deference to a study conducted in Gojjam in northwest Ethiopia (19.9%) [6], which was carried out in a similar setup/rural residents. However, due to the differences in the study area and socio-cultural conditions, it is noteworthy to mention that the direct comparison of our results with previous investigations employed in Ethiopia is impossible. Furthermore, the possible reason for the discrepancy might be due to the difference in methods and measures applied to assess dietary practice since this study used four measures; DDS, FVS, consumption of ASF, and frequency of meals over 1 week. However, most of the previous studies carried out in Ethiopia used meal frequency to evaluate the dietary practice of pregnant women, which could not comprehensively assess dietary practices.

Dietary diversity is correlated with the probability of nutrient adequacy, increased nutrient intake, and better nutritional status of pregnant women [30]. Evidence suggested that consumption of animal source foods guarantees the intake of micronutrients among pregnant women in developing countries [24, 31]. Although consumption of animal source food has a significant share of dietary quality, the practice decreases in low-income counties [25]. We observed that only 29.46% of pregnant women have a high dietary diversity score in the present study. However, 37.5% of participants have a high mean food variety score. Furthermore, 24.78% of the women consumed animal source foods 1 week before the survey. The current dietary diversity in pregnancy is lower than various studies conducted in Ethiopia [5, 29, 32, 33] but higher than the study carried out in Shashemane town, central Ethiopia [11].

Similar to reports from Shashamane [11] and Egypt [34], our results also found that the occupational status of pregnant women had a significant association with dietary practice. Pregnant women who were merchants had higher dietary practices. Housewives are more confined to household work and financially dependent on their family and partners than those merchants [35]. This could be because women who participated in yielding their family financial income have a better chance of earning income and better access to diversified foods and appropriate diets. This suggests that a program to improve women’s nutrition would be strengthened by increasing women’s involvement in family income generation, decision-making power, and economic independence.

The current study also revealed that women whose partners’ education level of high school and above had higher appropriate dietary practice, which is consistent with the former studies done in Wachamo [36], and Shashamane [11]. This might be because literate partners have a better understanding of the importance of consuming a quality diet in pregnancy, and they may positively influence their wives to have appropriate dietary practices. Pregnant women who received dietary guidance are expected to consume a diversified diet compared to those who do not receive nutritional advice.

In this study, pregnant women who frequently avoided food intake were negatively associated with appropriate dietary practices, which is in line with a study carried out in Nepal [35], and China [37]. Pregnant women who practiced food avoidance have a higher odds of poor dietary practice. Our results indicated that the pattern of food avoidance could lead to a decline in dietary consumption and then micronutrient deficiency. The possible rationale of the deference might be mainly in the present study; most of the pregnant women who did avoid food intake did so in the third trimester of their pregnancy and at the time of interview.

Although chewing khat is an intensively disseminating act in Ethiopia and developed countries, comprising America and Europe [37–43], health consequences are well understood. The result of the current study highlights the importance of reducing the high level of inappropriate dietary practice during pregnancy with proper interventions. Therefore, pregnant women should frequently be advised of the negative consequences of chat chewing and supported to improve their dietary consumption in pregnancy.

The strengths of this study include the following: validated food frequency questionnaires were used to assess dietary practice, food items were established based on consultation of key informants from the study area who were knowledgeable about the culture, local language, and locally consumed foods. Various limitations to be considered when interpreting our results include the following: the cross-sectional nature of the data limits causal inference between dietary practice and their correspondences and dietary diversity and correlates, and due to sample collection being from a single season, this limits the generalizability of the results to other reasons. In addition, due to individual differences of dietary consumption in the study setup over 7 days, we establish our definition of the reference period of 7 days. Women who could have eaten food items more than one in 7 days were also tagged with those who consumed one time over 7 days could underrate the amount used up is another limitation.

## Conclusion

The study showed that the dietary practice of pregnant women in the Haramaya district is sub-optimal. It can be concluded that greater access to resources through the involvement of women in yielding their family financial income, partners education, dietary restriction, and chewing chat, as gauged by the study, was noted to be significantly associated with dietary practice during pregnancy. Undesirable dietary habits and nutrition-related practices, which are often based on insufficient knowledge, traditions and taboos or poor understanding of the relationship between diet and health, can adversely affect nutritional status. Thus, we suggest that nutrition policy, programs, and interventions encourage prenatal dietary practice focusing on women’s empowerment in generating their family income and raising partners’ awareness of the benefit of the quality diet in pregnancy for mothers and newborns. Promoting husbands’ engagement in providing continuous care in pregnancy should be tailored to meet the need of pregnant women and to improve their dietary practice. Social and behavioral change communication on maternal nutrition should promote shifts in social norms on food taboos using religious leaders and influential community members to realize adequate nutrition for pregnant women.

## Supplementary Information

**Additional file 1.**

## Data Availability

All data are availability within the manuscript. Additional data can be obtained from the corresponding author on a reasonable request.

## Abbreviations

ANC: Antenatal Care
ASF: Animal Source Food
DDS: Dietary Diversity Score
EDHS: Ethiopian Demographic and Health Survey
FCS: Food Consumption Score
FVS: Food Variety Score
HDS-HRC: Health Demographic Surveillance and Health Research Center
PCA: Principal Component Analysis
WHO: World Health Organization
