# Supplementary material for: Prevalence and determinants of dietary practices among pregnant women in eastern Ethiopia
Source: BMC Nutr. 2022 Jan 11;8:3. doi: 10.1186/s40795-021-00494-4 (PMC8751267; doi:10.1186/s40795-021-00494-4)
Supplement: Supplementary file 1 — Additional file 1. [file 40795_2021_494_MOESM1_ESM.docx]

Table 1: Out put of principal components/correlation PCA of wealth index of pregnant women in Haramaya District, eastern Ethiopia, 2021 (n=448)

Rotation: (unrotated = principal)

Component Eigenvalue Difference Proportion Cumulative

Comp1 2.71937 .802237 0.1700 0.1700

Comp2 1.91714 .397026 0.1198 0.2898

Comp3 1.52011 .161108 0.0950 0.3848

Comp4 1.359 .0577646 0.0849 0.4697

Comp5 1.30124 .298139 0.0813 0.5511

Comp6 1.0031 .0553429 0.0627 0.6137

Comp7 .947757 .0429759 0.0592 0.6730

Comp8 .904781 .0681784 0.0565 0.7295

Comp9 .836602 .0184705 0.0523 0.7818

Comp10 .818132 .0322204 0.0511 0.8330

Comp11 .785911 .0796702 0.0491 0.8821

Comp12 .706241 .163776 0.0441 0.9262

Comp13 .542465 .0370978 0.0339 0.9601

Comp14 .505368 .436151 0.0316 0.9917

Comp15 .0692169 .00565737 0.0043 0.9960

Comp16 .0635595 . 0.0040 1.0000

Rotation: orthogonal varimax (Kaiser off)

Component Variance Difference Proportion Cumulative

Comp1 2.2132 .362407 0.1383 0.1383

Comp2 1.85079 .223212 0.1157 0.2540

Comp3 1.62758 .156885 0.1017 0.3557

Comp4 1.47069 .0571941 0.0919 0.4476

Comp5 1.4135 .169293 0.0883 0.5360

Comp6 1.24421 . 0.0778 0.6137

Component rotation matrix

Comp1 Comp2 Comp3 Comp4 Comp5 Comp6

Comp1 0.7499 0.5629 0.2892 0.1023 -0.1536 0.0554

Comp2 -0.4131 0.3168 0.5347 0.2244 0.4559 0.4301

Comp3 0.2330 -0.5244 0.1177 0.8090 -0.0063 0.0485

Comp4 0.0890 -0.3886 0.7442 -0.3620 0.0280 -0.3943

Comp5 0.4416 -0.2326 -0.2269 -0.2500 0.7763 0.1852

Comp6 0.0991 -0.3203 0.1064 -0.3020 -0.4064 0.7873
